# Expression of Livin in Colorectal Cancer and Its Relationship to Tumor Cell Behavior and Prognosis

**DOI:** 10.1371/journal.pone.0073262

**Published:** 2013-09-02

**Authors:** Dae-Seong Myung, Young-Lan Park, Cho-Yun Chung, Hyung-Chul Park, Jong-Sun Kim, Sung-Bum Cho, Wan-Sik Lee, Kyung-Hwa Lee, Jae-Hyuk Lee, Young-Eun Joo

**Affiliations:** 1 Departments of Internal Medicine, Chonnam National University Medical School, Gwangju, Republic of Korea; 2 Departments of Pathology, Chonnam National University Medical School, Gwangju, Republic of Korea; Vanderbilt University Medical Center, United States of America

## Abstract

**Backgrounds:**

Expression of Livin, a member of the inhibitors of apoptosis protein family, is associated with tumor development and progression. The aims of this study were to evaluate whether Livin affects oncogenic biological behavior of colorectal cancer cells, and to document the relationship between its expression and various clinicopathological parameters in colorectal cancer.

**Methods:**

We investigated the impact of Livin on tumor cell behavior by using the small interfering RNA and pcDNA3.1 vector in SW480 and DKO1 colorectal cancer cell lines. The expression of Livin was investigated by RT-PCR and immunohistochemistry in coloretcal cancer tissues. The apoptotic cells were visualized by TUNEL assay, and proliferative cells were visualized by Ki-67 antibody staining.

**Results:**

Knockdown of Livin suppressed tumor cell migration and invasion in colorectal cancer cells. Knockdown of Livin induced the apoptosis by up-regulating of caspase-3, -7 and PARP activities and the cell cycle arrest by decreasing cyclin D1, cyclin D3, cyclin-dependent kinase 4 and 6, and by inducing p27 expression. The MAPK signaling cascades were significantly blocked by knockdown of Livin. In contrast, overexpression of Livin enhanced tumor cell migration and invasion, and inhibited the apoptosis and cell cycle arrest. The mean apoptotic index (AI) value of Livin positive tumors was significantly lower than AI of Livin negative tumors. However, there was no significant difference between Livin expression and Ki-67 labeling index (KI). Livin expression was significantly increased in colorectal cancer and metastatic lymph node tissues compared to normal colorectal mucosa and non-metastatic lymph node tissues and was associated with tumor stage, lymphovascular invasion, lymph node metastasis and poor survival.

**Conclusions:**

These results indicate that Livin is associated with tumor progression by increasing tumor cell motility and inhibiting apoptosis in colorectal cancer.

## Introduction

Colorectal cancer is one of the leading causes of cancer-associated morbidity and mortality in the world. Despite evidence that 5-year survival is 90% when colorectal cancer is diagnosed at an early stage, < 40% of cases are diagnosed when the cancer is still localized [1]. Rapid advances in our understanding about the molecular and biologic characteristics of colorectal cancer have provided useful knowledge into the pathogenesis of colorectal cancer. Biomarkers have been developed to identifying individuals who will benefit most from cancer surveillance and management [2-5]. Identifiying biomarkers that can detect colorectal cancer earlier or monitor cancer progression would enable personalization of medicine and improve survival rates of patients with cancer.

The underlying mechanisms of action in cancer progression are beginning to be unraveled. The reported molecular and biochemical mechanisms that may contribute to the phenotypic changes in favor of carcinogenesis, include inhibited apoptosis, enhanced tumor cell proliferation, increased invasiveness, perturbation of cell adhesion, promotion of angiogenesis, and inhibited immune surveillance. These events may contribute to the development and progression of cancer [6-8].

Apoptosis plays an important role in many biological events, including morphogenesis, cell turnover and elimination of harmful cells. A disturbance in apoptosis may confer a survival advantage on malignant cells harboring genetic alterations and thus promote cancer progression [9,10]. The central event in apoptosis is the proteolytic activation of a class of cysteine aspartyl-specific proteases, the caspases. Initiator caspases cleave effector caspases which in turn degrade a number of intracellular protein substrates and thereby induce the characteristic morphological hallmarks of apoptosis [11]. These caspase activities are inhibited by the inhibitors of apoptosis proteins (IAPs) family. Until now, eight human IAPs have been identified, including c-IAP1, c-IAP2, NAIP, XIAP, ILP-2, BRUCE, Survivin and Livin [12]. Livin was recently identified to be a novel anti-apoptotic gene. Livin is recruited to death receptor signaling complexes, where it inhibits activation of caspases responsible for apoptosis and protects cells from diverse pro-apoptotic stimuli. Livin is associated with the induction of oncogenic phenotypes including invasion, motility, cell proliferation and inhibition of apoptosis in human cancer cell lines [13-16]. Additionally, Livin expression in the vast majority of human cancers is enhanced and correlated with cancer development and progression [17-22]. Silencing of the Livin gene using small interfering RNA (siRNA) decreases tumor volume by inducing apoptosis in a xenograft model of colorectal cancer [23]. Therefore, Livin is considered a potential therapeutic target for treating colorectal cancer.

The aims of this study were to evaluate whether Livin affects oncogenic biologic behavior of human colorectal cancer cells, to evaluate Livin expression in human colorectal cancer tissues, and to examine the correlation of Livin with apoptosis, tumor cell proliferation and clinicopathological features including survival.

## Materials and Methods

### Ethics statement

This study was approved by the Institutional Review Board of Chonnam National University Hwasun Hospital (Jeonnam, Korea). A written informed consent was obtained from each participant prior to tissue acquisition. All participants gave written consent of their information to be stored in the hospital database and used for research.

### Patients and tissue samples

Twenty colorectal cancer tissues and paired normal colon tissues were collected by colonoscopic biopsy for RNA and protein preparations at Chonnam National University Hwasun Hospital. Formalin-fixed and paraffin-embedded tissue samples from 161 randomly chosen patients who had undergone surgery for colorectal cancer at Chonnam National University Hwasun Hospital between January 2004 and December 2004 were obtained for immunohistochemistry. No patient had undergone preoperative radiotherapy or chemotherapy. Pathological reports and clinical histories at the time of surgery were reviewed in medical records. Tissue blocks were selected by viewing original pathological slides and choosing blocks that showed the junction between normal colon epithelium and the tumor region. Tumors were staged in accordance with the American Joint Committee on Cancer staging system [24]. Survival was measured from the time of surgery until follow-up on December 31, 2010.

### Cell culture and siRNA transfection

The SW480 and DKO1 Human colorectal cancer cell lines were obtained from the American Type Culture Collection (Rockville, MD, USA) and cultured in DMEM (Hyclone, Loan, UT, USA) supplemented with 10% fetal bovine serum (Hyclone) in a humidified incubator at 37℃ with a 5% CO_2_ atmosphere. Livin and scrambled siRNA were purchased from Santa Cruz Biotechnology (Santa Cruz, CA, USA) and Qiagen (Valencia, CA, USA), respectively. Livin cDNA was subcloned into pcDNA3.1 vector (Invitogen, Carlsbad, CA, USA). Livin construction was verified by sequencing. The specific gene were transfected using Lipofectamine^TM^ RNAiMAX and Lipofectamine^TM^ 2000 (Invitrogen) according to the manufacturer’s recommendations and then incubated for 48 h. In addition, cells transfected with pcDNA3.1 vector were selectively treated with 5-Fluorouracil (5-FU) (10μg/ml, Choong-Wae, Chung-Nam, Korea) for 48 h.

### Reverse transcription-polymerase chain reaction (RT-PCR)

Total RNA was extracted using Trizol reagent (Invitrogen) and reverse-transcribed with MMLV transcription reagents (Invitrogen) according to the manufacturer’s recommendations. PCR amplification of cDNA was performed using gene-specific primers and Go Taq^®^ DNA polymerase (Promega, Madison, WI, USA). The following specific primers were used; Livin 5’-CACACAGGCCATCAGGACAAG-3’/5’-ACGGCACAAAGACGATGGAC-3’; GAPDH 5'-ACCACAGTCCATGCCATCAC-3'/ 5'-TCCACCACCCTGTTGCTGTA-3'.

### Western blotting

Cell lysates were prepared using M-PER^®^ Mammalian Protein Extraction reagent (Thermo, Rockford, IL, USA) with Halt^TM^ Phosphatase inhibitor and Halt^TM^ Protease inhibitor cocktail (Thermo). Total proteins were electrotransferred onto PVDF membranes (Millipore, Billerica, MA, USA), and the specific proteins were blotted with primary antibody. The following antibodies were used: antibodies against Livin, X-chromosome binding IAP (XIAP), Survivin and β-tubulin were purchased from Santa Cruz Biotechnology (Santa Cruz, CA, USA). Antibodies against extracellular signal-regulated kinase (ERK), phospho-ERK, p38, and phospho-p38, c-Jun NH_2_-terminal kinase (JNK), phospho-JNK, phospho-Akt, Akt, phospho-p65, cleaved caspase (3, 7, and 9), cleaved poly (ADP-ribose) polymerase (PARP), cyclin-dependent kinase 4 (CDK4), CDK6, cyclin D1, cyclin D3, cyclin B1, p21, p27, p57, p15, p16 and second mitochondria-derived activator of caspases/direct IAP binding protein with low pI (SMAC/DIABLO) were purchased from Cell Signaling Technology (Danvers, MA, USA). Immunoreactive proteins were visualized by the enhanced chemiluminescence detection system HRP substrate (Millipore) and the LAS-4000 luminescent image analyzer (Fujifilm, Tokyo, Japan).

### Cell invasion assay

Invasive ability was calculated by the number of cells that passed through Transwell filter chambers (Corning Inc., Corning, NY, USA) with 8 μm pores. Transwell filters were coated with 1% gelatin overnight and dried at room temperature (RT). Cells were seeded on viable cells of 2 x 10^5^ in 0.2% BSA medium in the upper chamber. Human plasma fibronectin (Calbiochem, La Jolla, CA, USA) was added as a chemoattractant to 0.2% BSA medium in the lower chamber. After a 24 h incubation, the invaded cells on the bottom surface of the Transwell were stained with Diff-Quik solution (Sysmex, Kobe, Japan) and counted in five selected fields under a light microscope. Data are expressed as mean±standard deviation of the number of cells/field in three individual experiments.

### Cell migration assay

The cell migration assay was performed using Culture-Inserts (2 x 0.22 cm^2^; Ibidi, Regensburg, Germany). To create a wound gap, cells were seeded in the Culture-Inserts, which were gently removed after a 24 h incubation using sterile tweezers. The progress of wound closure was photographed with an inverted microscope. The distance between gaps was normalized to 1 cm after capture from three random sites.

### Cell viability

Cell viability was determined with the EZ-CyTox (tetrazolium salts, WST-1) cell viability assay kit (Daeil Lab Inc., Seoul, Korea). After applying the WST-1 reagent at 37℃, cell viability was measured using a microplate reader (Infinite M200, Tecan, Austria GmbH, Vienna, Austria) with Magellan V6 data analysis software (Tecan). Triplicate wells were used for each experiment and all experiments were conducted at least in triplicate.

### Flow cytometric analysis

Transfected cells were trypsinized, collected in PBS, and resuspended in 1x binding buffer (BD Biosciences, San Diego, CA, USA). Cell suspensions were incubated in APC Annexin V and 7-amino-actinomycin D (BD Biosciences) at RT. For the cell cycle analysis, the cells were incubated in 10 μg/ml ribonuclease A (Sigma-Aldrich, St. Louis, MO, USA) and 50 μg/ml propidium iodide (PI) at RT in the dark. The population of Annexin-V-positive cells and the cell cycle phase were analyzed using a BD Cell Quest^®^ version 3.3 instrument (Becton Dickinson, San José, CA, USA) and WinMDI version 2.9 software (The Scripps Research Institute, San Diego, CA, USA).

### Immunohistochemistry

Paraffin tissue sections from patients were deparaffinized, rehydrated and retrieved with retrieval buffer. The tissues were treated with a Peroxidase-Blocking solution (Dako, Carpinteria, CA, USA) to block the endogenous peroxidase activity and were incubated with polyclonal rabbit anti-human Livin in primary diluent solution (Invitrogen) overnight at 4°C. After washing in TBST, the tissues were stained using Dako, Real^TM^ Envision HRP/DAB detection system (Dako). Stained tissues were viewed and photographed under a light microscope.

### Evaluation of Livin expression

Immunostained specimens were evaluated independently by two observers without knowledge of the clinicopathological data. If there was a discrepancy, a consensus was reached after further evaluation. The intensity of positive cancer cells was graded on a scale of four: 0, no staining of cancer cells; 1, weak staining; 2, moderate staining; 3, strong staining. The percentage of stained cancer cells was also graded on a scale of four: 0, none; 1, <10%; 2, 10-50%; 3, >50%. The intensity rating was multiplied by the percent stain rating to obtain an overall score. The mean overall score for the 161 tumors analyzed was 4.0. Thus, the mean overall score of 4.0 was chosen as the cut-off point for discriminating Livin expression status. Specimens with a score >4 were regarded as positive, and those with a score ≤ 4 were regarded as negative expression.

### Assessment of tumor cell proliferation

Proliferating tumor cells were visualized by staining immunohistochemically using an anti-Ki-67 antibody (MIB-1; diluted 1:150; Dakopatts, Glostrup, Denmark). Distinct nuclear Ki-67 immunoreactivity was considered positive. The Ki-67 labeling index (KI) is presented as the number of Ki-67-positive nuclei/1000 tumor cell nuclei. KI has been used to estimate the proliferative ability of tumor cells.

### Terminal deoxynucleotidyl transferase (TdT)-mediated deoxyuridine triphosphate (dUTP) nick end labeling (TUNEL) assay

Apoptotic cells and bodies were detected using the DeadEnd^TM^ Colorimetric TUNEL System (Promega, Madison, WI, USA), according to the manufacturer’s protocol. Briefly, tissue sections were dewaxed and hydrated through a graded alcohol series. Permeabilization was then performed in Proteinase K solution for 15 min. Labeling was performed by adding the enzyme TdT reaction mix to tissue sections on the slides for 60 min in a 37℃ humidified chamber. Labeled cells were developed using streptavidin HRP and 3,3-diaminobenzidine enzyme substrate. A quantitative method for calculating apoptotic cells was used by two independent pathologists. All sections were examined under high power fields (magnification of 40 x 10). The fields were selected in the highest labeled area for each case. The apoptotic index (AI) was expressed as the number of positive nuclei including apoptotic bodies among 1000 tumor cell nuclei.

### Statistical analysis

Data from at least three independent experiments were used to compare between groups. Data are presented as means ± standard deviations. Student’s *t*-test was used to determine statistical significance for the intergroup comparisons. The χ^2^-test and Fisher’s exact test, where appropriate, were used to compare Livin expression with various clinicopathological parameters. The relationships between Livin expression and the KI or AI were evaluated by the Student’s *t*-test. Actuarial survival rates of patients with positive or negative Livin expression were evaluated according to the Kaplan-Meier method, and the differences were tested with a log-rank test. The Statistical Package for the Social Sciences (SPSS/PC+ 15.0, Chicago, IL, USA) was used for all analyses. A p-value < 0.05 was considered significant.

## Results

### The impact of Livin on invasion, migration and proliferation of human colorectal cancer cells

**Figure 1:**
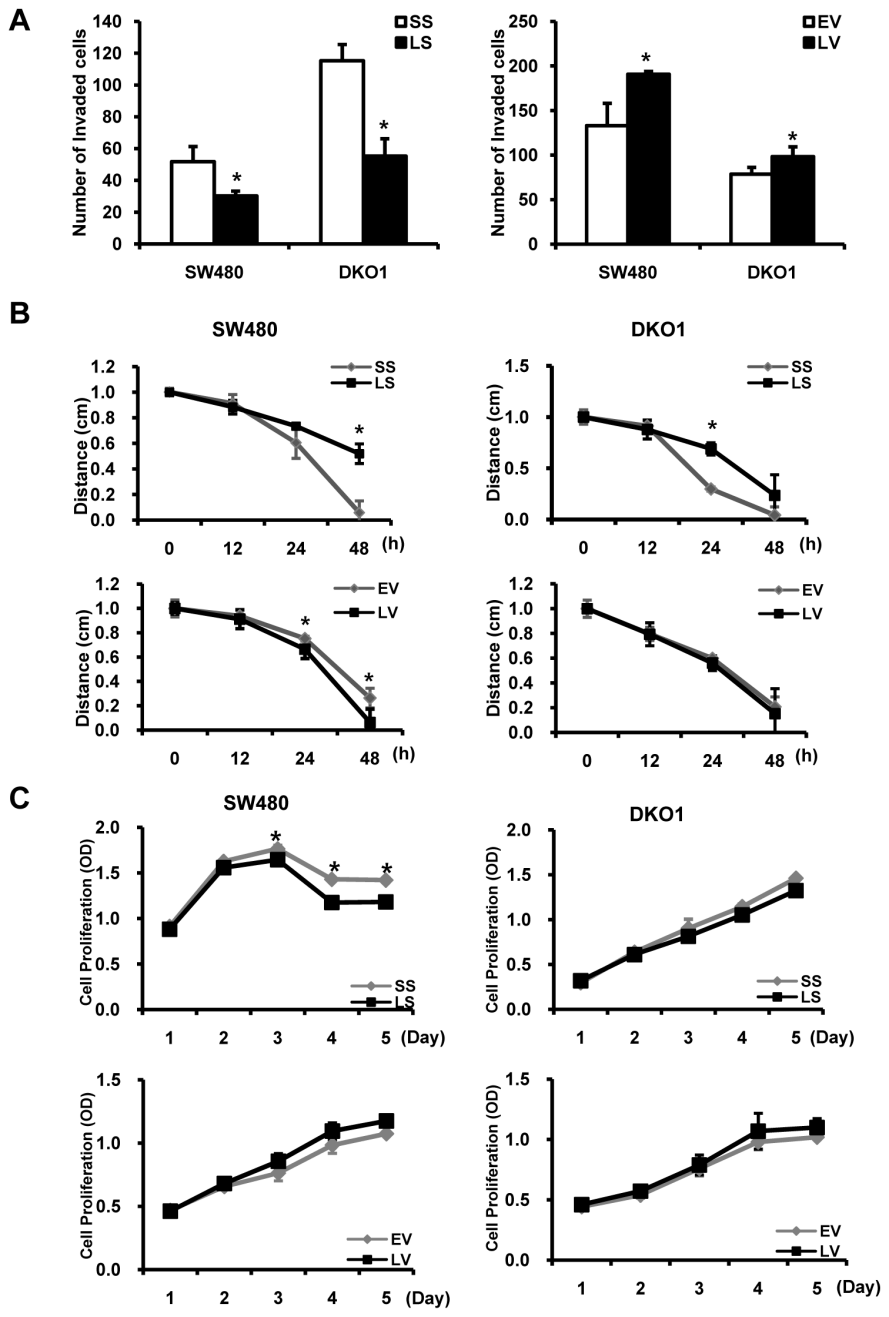
The impact of Livin on invasion, migration and proliferation of human colorectal cancer cells. (A) The impact of Livin on invasion of colorectal cancer cells. The invasion assay using the siRNA or pcDNA3.1-transfected cells was performed. Stained invading cells were counted and are represented as a graph between groups. The number of LS-transfected cells that invaded was significantly lower than that of SS-transfected cells. The number of invading cells was significantly higher in LV-transfected cells compared to EV-transfected cells (mean±standard error [SE], n=6; *p<0.05). (B) The impact of Livin on migration of colorectal cancer cells. The wound healing assay using siRNA or pcDNA3.1-transfected cells was performed and graphs of cell migration are displayed as relative healing distances. Cell migration was significantly disturbed in LS-transfected SW480 and DKO1 cells and increased in LV-transfected SW480 cells (mean±SE, n=3; *p<0.05). (C) The impact of the Livin on proliferation of colorectal cancer cells. The absorbance indicating proliferating viable cells decreased in the LS-transfected SW480 cells but there was no significant difference of cell proliferation between LV and EV transfected cells (mean±SE, n=3; *p<0.05). SS; scramble siRNA, LS; Livin siRNA, EV; Empty-pcDNA3.1, LV; pcDNA3.1-Livin.

To investigate the function of Livin on oncogenic biological behavior of colorectal cancer cells, Livin siRNA or pcDNA3.1-Livin was used to control the endogenous Livin gene expression in SW480 and DKO1 human colorectal cancer cell lines. Livin gene expression in all tested cells, showed a specific decrease at mRNA and protein levels by transfection of Livin siRNA and a specific increase by transfection of pcDNA3.1-Livin. The numbers of invading Livin siRNA-transfected SW480 and DKO1 cells were 30.2±3.1 and 55.3±11.0, whereas 51.8 ± 9.5 and 115.3 ± 10.3 cells were observed for scrambled siRNA-transfected cells. Cells were measured in six random 0.5 × 0.5 mm^2^ microscopic fields using 10 μg/ml fibronectin as a chemoattractant. The difference between the two cell types was significant (p=0.015 and p<0.001, respectively). In contrast, the number of invading cells was significantly increased in pcDNA3.1-Livin transfected SW480 and DKO1 cells compared to empty-pcDNA3.1 transfected cells (p=0.024 and p=0.012, respectively) (Figure 1A). The artificial wound gap in plates of scrambled siRNA-transfected cells became significantly narrower than that in Livin siRNA-transfected cells at 48 h in SW480 and at 24 h in DKO1 cells (p=0.012 and p=0.016, respectively). The artificial wound gap in pcDNA3.1-Livin transfected SW480 cells became significantly narrower than that in empty-pcDNA3.1 transfected cells at 24 and 48 h (p=0.034 and p=0.008, respectively) (Figure 1B). The cell proliferation assay was performed at 2, 3, 4, and 5 days after transfection of Livin siRNA or pcDNA3.1-Livin to access the potential of Livin on cell proliferation. Proliferating cells, as determined by absorbance, decreased significantly in the Livin siRNA-transfected SW480 cells compared to the scrambled siRNA-transfected cells at 3, 4 and 5 days (p=0.005, <0.001, and 0.022, respectively), but no significant difference was observed in DKO1 cells. Additionally, in all tested cells, there was no significant difference of cell proliferation between pcDNA3.1-Livin and empty-pcDNA3.1 transfected cells (Figure 1C).

### The impact of Livin on apoptosis in human colorectal cancer cells

**Figure 2:**
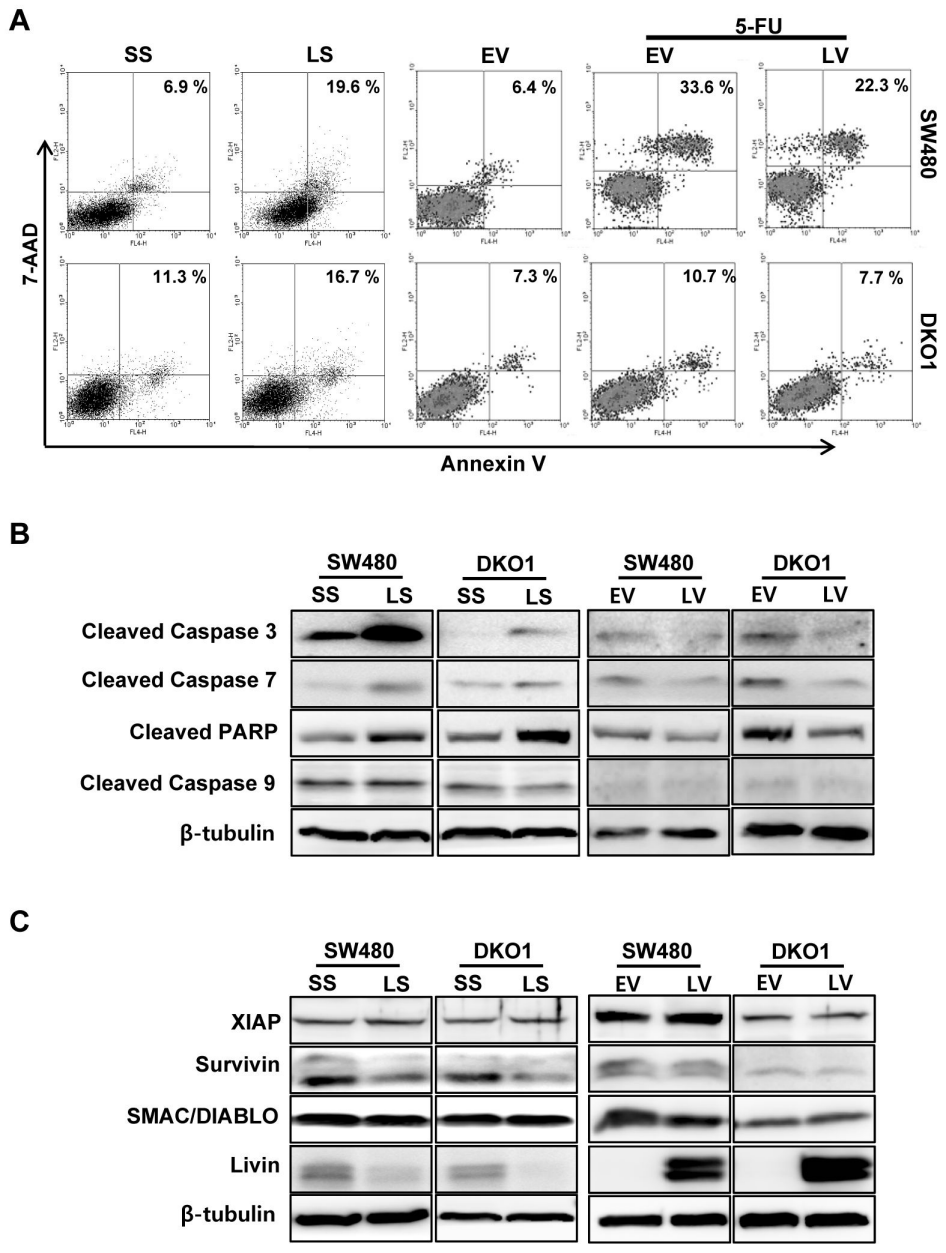
The impact of Livin on apoptosis in human colorectal cancer cells. (A) The proportion of apoptotic cells induced by transfection of LS was greater than that induced by transfection of SS (6.9 vs. 19.6%) in SW480 cells but Livin knockdown had a minimal influence on apoptosis (11.3 vs. 16.7%) in DKO1 cells. Overexpression of Livin by transfection of LV inhibited the apoptosis of SW480 cells in response to 5-FU but overexpression of Livin had a minimal influence on apoptosis in DKO1 cells (B) Expression of cleaved caspase-3, -7, -9, and PARP proteins. The caspase-3, -7 and PARP expression was increased in SW480 and DKO1 cells after Livin knockdown, and decreased after overexpression of Livin (C) Expression of apoptosis regulatory proteins. Survivin protein level decreased following Livin knockdown in SW480 and DKO1 cells, but XIAP and SMAC/DIABLO protein levels were not altered in response to Livin knockdown. Additionally, Survivin, XIAP and SMAC/DIABLO protein levels were not altered after overexpression of Livin. PARP; Poly (ADP-ribose) polymerase, XIAP; X-chromosome binding IAP, SMAC/DIABLO; second mitochondria-derived activator of caspases/direct IAP binding protein with low pI, SS; scramble siRNA, LS; Livin siRNA, EV; Empty-pcDNA3.1, LV; pcDNA3.1-Livin, 5-FU; 5-fluorouracil, 7-AAD; 7-amino-actinomycin D.

We performed flow cytometric analyses to evaluate the impact of Livin on apoptosis. The cell apoptotic rate induced by transfection of Livin siRNA increased significantly, compared with that induced by transfection of scrambled siRNA (6.9 *vs.* 19.6%) in SW480 cells but Livin knockdown had a minimal influence on apoptosis (11.3 *vs.* 16.7%) in DKO1 cells (Figure 2A). 5-FU is well known to induce apoptosis and affect cell cycle of the cancer cells. Overexpression of Livin by transfection of pcDNA3.1-Livin inhibited the apoptosis of SW480 cells in response to 5-FU, but overexpression of Livin had a minimal influence on apoptosis in DKO1 cells (Figure 2A). We further investigated caspase-specific activities to determine the activation of caspases during knockdown and overexpression of Livin. Cleaved caspases-3 and -7 and PARP expressions were up-regulated in the SW480 and DKO1 cells after Livin knockdown and down-regulated after overexpression of Livin (Figure 2B). As shown in Figure 2C, the Survivin protein level was reduced due to Livin knockdown in SW480 and DKO1 cells, but XIAP and SMAC/DIABLO protein levels were not altered in response to Livin knockdown. Additionally, the Survivin, XIAP and SMAC/DIABLO protein levels were not altered after overexpression of Livin.

### The impact of Livin on cell cycle arrest in human colorectal cancer cells

**Figure 3:**
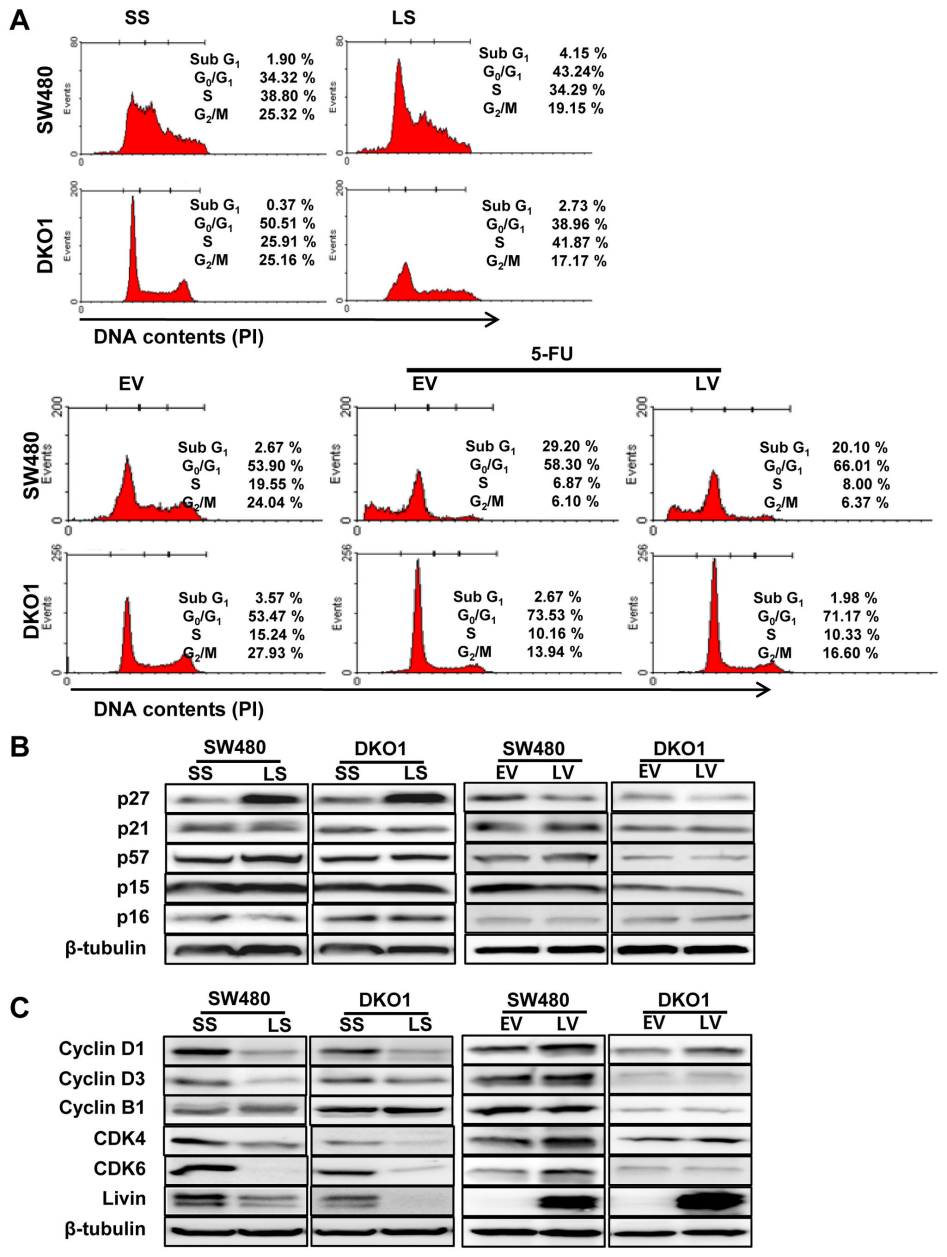
The impact of Livin on cell cycle arrest in human colorectal cancer cells. (A) Livin knockdown resulted in cell cycle arrest in the G0/G1 phase of SW480 cells and the S phase of DKO1 cells. 5-FU treatment induced cell cycle arrest in the subG1 phase of SW480 and the G0/G1 phase of DKO1 cells. Overexpression of Livin inhibited 5-FU-induced cell cycle arrest in SW480 cells and had a minimal influence in DKO1 cells. One representative experiment of the three independent experiments is shown. (B) Expression of cyclin-dependent kinase (CDK) inhibitor proteins. The p27 protein level was significantly increased by Livin knockdown and decreased by overexpression of Livin in SW480 and DKO1 cells. However, p21, p57, p15 and p16 protein levels were not altered in response to knockdown or overexpression of Livin. (C) Expression of cyclins and cyclin-dependent kinase (CDKs) proteins. Cyclin D1, cyclin D3, CDK4 and CDK6 protein levels were significantly decreased by Livin knockdown and increased the cyclin D1 and CDK4 by overexpression of Livin in SW480 and DKO1 cells. SS; scramble siRNA, LS; Livin siRNA, EV; Empty-pcDNA3.1, LV; pcDNA3.1-Livin.

We performed flow cytometric analyses to detect whether Livin could change cell cycle distribution. Livin knockdown resulted in cell cycle arrest in the G0/G1 phase of SW480 cells and the S phase of DKO1 cells (Figure 3A). 5-FU treatment induced cell cycle arrest in the subG1 phase of SW480 and G0/G1 phase of DKO1 cells. Overexpression of Livin inhibited 5-FU-induced cell cycle arrest in SW480 cells and had a minimal influence in DKO1 cells (Figure 3A). Next, we evaluated the effects of Livin on various CDK inhibitors (CDKIs) involved in cell cycle arrest in human colorectal cancer cells. As shown in Figure 3B, the p27 protein level increased significantly by Livin knockdown, and decreased by overexpression of Livin in SW480 and DKO1 cells. However, p21, p57, p15 and p16 protein levels were not altered in response to knockdown and overexpression of Livin. Cyclins and CDKs are negatively regulated by CDKIs. Therefore, we examined the effect of Livin on expressed levels of cyclin D1, cyclin D3, cyclin B1, CDK4, and CDK6. As shown in Figure 3C, the cyclin D1, cyclin D3, CDK4, and CDK6 protein levels were significantly decreased by Livin knockdown, and increased the cyclin D1 and CDK4 by overexpression of Livin in SW480 and DKO1 cells.

### The impact of Livin on intracellular signaling pathways involved in apoptosis and cell cycle arrest in human colorectal cancer cells

**Figure 4:**
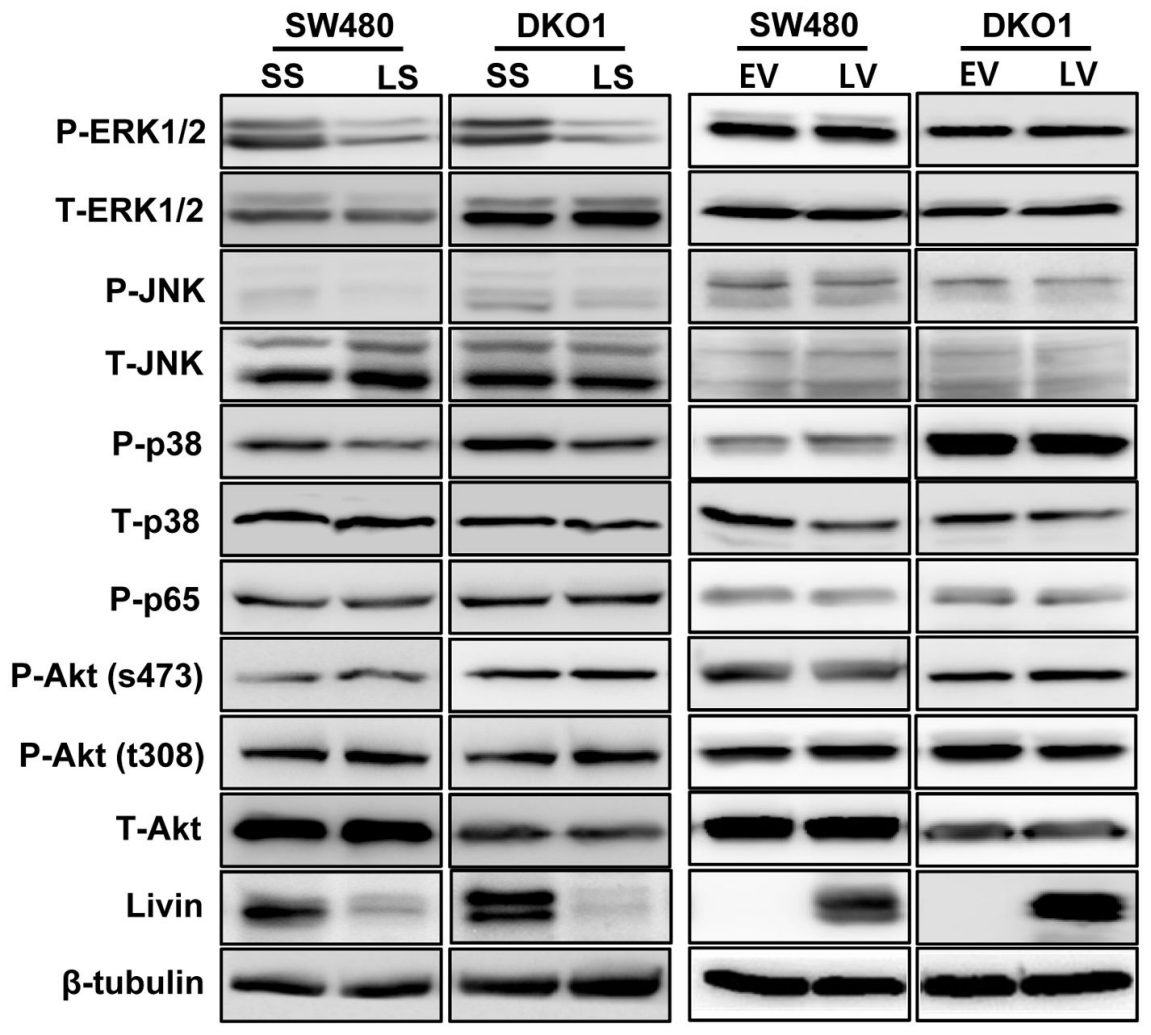
The impact of Livin on intracellular signaling pathways involved in apoptosis and cell cycle arrest of human colorectal cancer cells. The phosphorylation levels of ERK1/2, JNK and p38 decreased following Livin knockdown of SW480 and DKO1 cells. But Akt and p65 the phosphorylation levels showed no change following Livin knockdown. Additionally, ERK1/2, JNK, p38, Akt and p65 phosphorylation levels were not changed by overexpression of Livin. SS; scramble siRNA, LS; Livin siRNA, EV; Empty-pcDNA3.1, LV; pcDNA3.1-Livin.

We studied the effect of Livin on stimulation of intracellular signaling pathways leading to apoptosis and cell cycle arrest in SW480 and DKO1 cells to explore the potential mechanisms involved in apoptosis and cell cycle arrest. The phosphorylation levels of ERK1/2, JNK and p38 were down-regulated in Livin knockdowned SW480 and DKO1 cells (Figure 4). But Akt and p65 phosphorylation levels remained unchanged by Livin knockdown. Additionally, ERK1/2, JNK, p38, Akt and p65 phosphorylation levels remained unchanged by overexpression of Livin.

### Livin expression in human colorectal cancer and metastatic lymph node tissues

**Figure 5:**
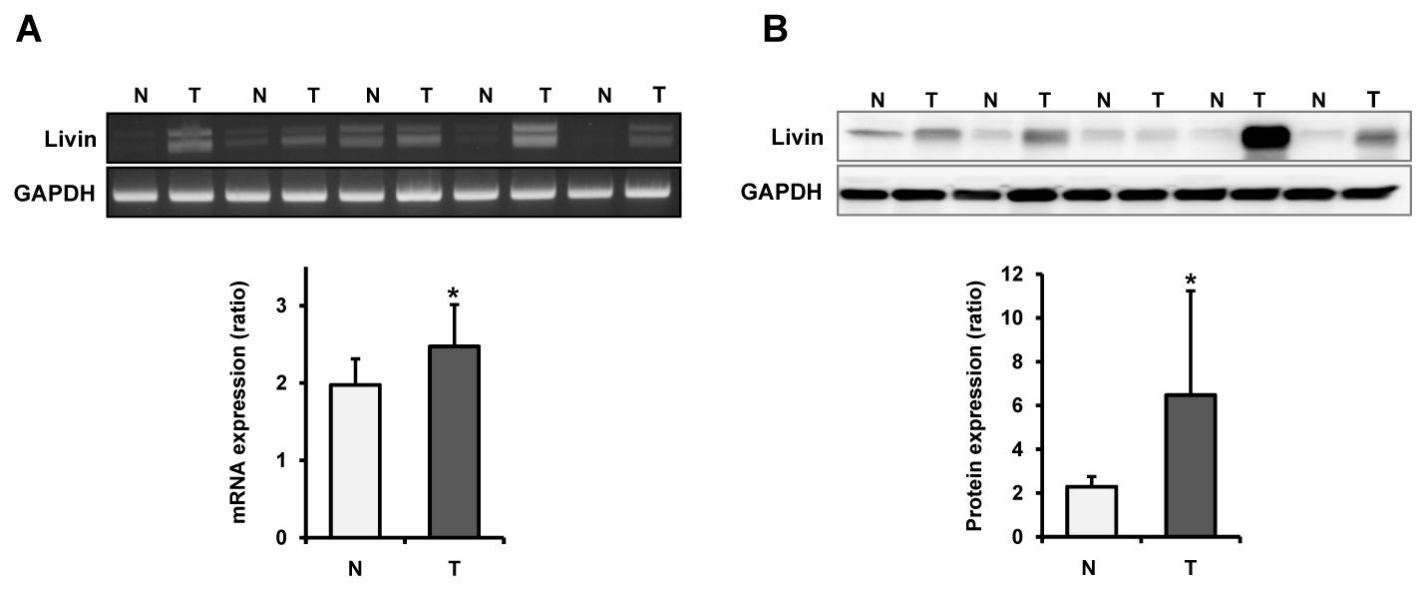
Expression of Livin mRNA and protein in fresh colonoscopic biopsy specimens. (A) Livin mRNA expression (B) Livin protein expression. Expression of Livin is upregulated in cancer tissues compared to paired normal mucosa at mRNA and protein levels in fresh colonoscopic biopsy specimens. Each bar represents the mean ± SE of 20 cases. *p<0.05 versus normal colorectal mucosa. T; colorectal cancer tissue, N; paired normal colorectal mucosa.

**Figure 6:**
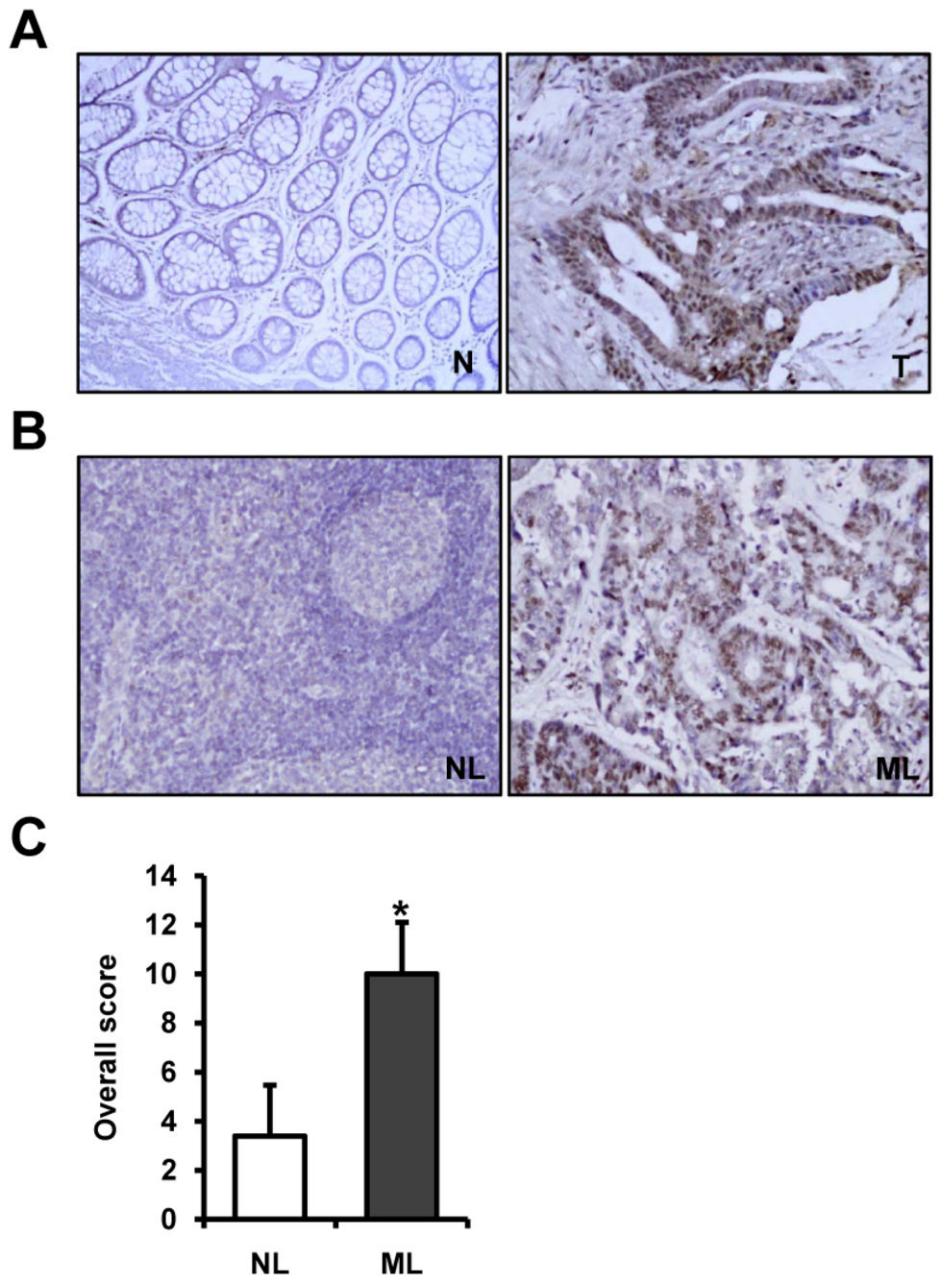
Livin protein expression in human colorectal cancer and metastatic lymph node tissues by immunohistochemistry. (A) The Livin protein was predominantly immunostained in the nuclei of cancer cells but did not or only weakly immunostained in normal colorectal mucosa (× 100). (B) The immunostaining of Livin in metastatic lymph node tissues was significantly stronger than that in non-metastatic lymph node tissues (× 100). (C) The overall score for Livin immunostaining in metastatic lymph node tissues was significantly higher than that in non-metastatic lymph node tissues (*p<0.001). T; colorectal cancer tissue, N; paired normal colorectal mucosa, NL; non-metastatic lymph node tissue, ML; metastatic lymph node tissue.

To confirm the results of the colorectal cancer cell line studies, we evaluated Livin expression at the RNA and protein levels by RT-PCR, Western blotting and immunohistochemistry in human colorectal cancer tissues, paired normal colorectal mucosa, and metastatic and non-metastatic lymph node tissues of same patients taken from colonoscopic biopsy and surgical specimens. In the colonoscopic biopsy specimens, we confirmed up-regulation of Livin expression in cancer tissues compared to that in paired normal mucosa at the RNA and protein levels (p=0.035 and p=0.020, respectively) (Figure 5A, B). In the paraffin tissue sections, the Livin protein did not or only weakly immunostained in normal colorectal mucosa (Figure 6A). Immunostaining of the Livin protein was predominantly identified in the nuclei of cancer cells but was not detectable in the tumor stroma (Figure 6A). Immunostaining of Livin in metastatic lymph node tissues was significantly stronger than that in non-metastatic lymph node tissues (Figure 6B). The overall score for Livin immunostaining in metastatic lymph node tissues was significantly higher than that in nonmetastatic lymph node tissues (*P*<0.001) (Figure 6C).

### Correlations between Livin expression and tumor cell proliferation and apoptosis in human colorectal cancers

**Figure 7:**
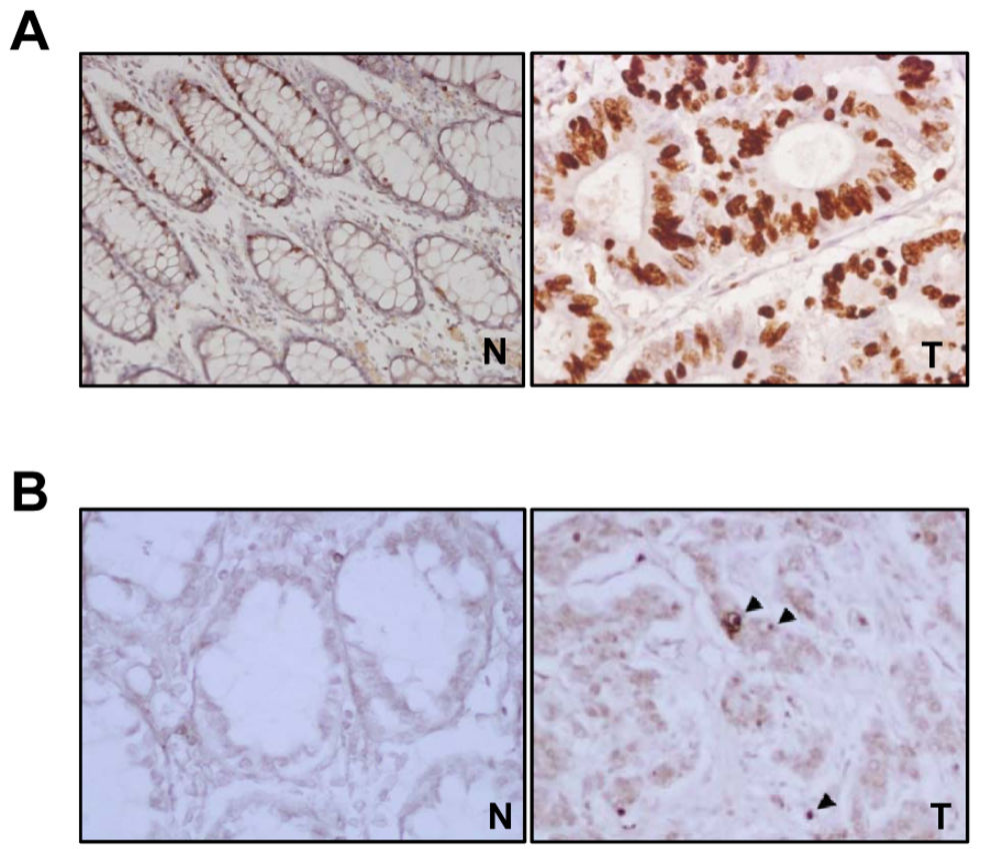
Assessment of tumor cell proliferation and apoptosis in human colorectal cancers. (A) Immunostaining of Ki-67. Immunostaining of Ki-67 shows strong nuclear positivity in the cancer cells but is rarely positive in the normal colorectal mucosa (× 200). (B) Detection of apoptotic cells (arrow) by TUNEL staining. Apoptotic cells with classic features of DNA condensation are shown to have a halo consisting of pyknotic nucleus and shrunken cytoplasm (arrow head) in colorectal cancer tissues but TUNEL staining is not positive in normal colorectal mucosa (× 400). TUNEL, terminal deoxynucleotidyl transferase (TdT)-mediated deoxyuridine triphosphate (dUTP) nick end labeling. T; colorectal cancer tissue, N; paired normal colorectal mucosa.

Ki-67 immunoreactivity was predominantly found in the nuclei of cancer cells (Figure 7A). The KI for 161 tumors ranged from 21.9 to 85.6 with a mean KI of 52.8±15.1. No significant difference was observed between Livin expression and KI (p=0.504) (Table 1). Standard morphological criteria for apoptotic cells using the TUNEL assay are the presence of beaded or shrunken chromatin and apoptotic bodies with a clear halo (Figure 7B). The AI for 161 tumors ranged from 0.6 to 30.0 with a mean AI of 8.8±5.6. The mean AI value of Livin-positive tumors was 6.4±3.7, which was significantly lower than the AI of Livin-negative tumors (p=0.009) (Table 1).

**Table 1:** Correlations between Livin expression and tumor cell proliferation and apoptosis in colorectal cancers.

Indices	Total (n=161)	Livin expression		p-value
		Negative (n=86)	Positive (n=75)	
KI (Mean±SD)	52.8±15.1	51.1±14.7	54.3±15.6	0.504
AI (Mean±SD)	8.8±5.6	11.3±6.4	6.4±3.7	0.009

KI, Ki-67 labeling index; AI, apoptotic index; SD, standard deviation

### Correlations between Livin expression and clinicopathological features in human colorectal cancers

**Figure 8:**
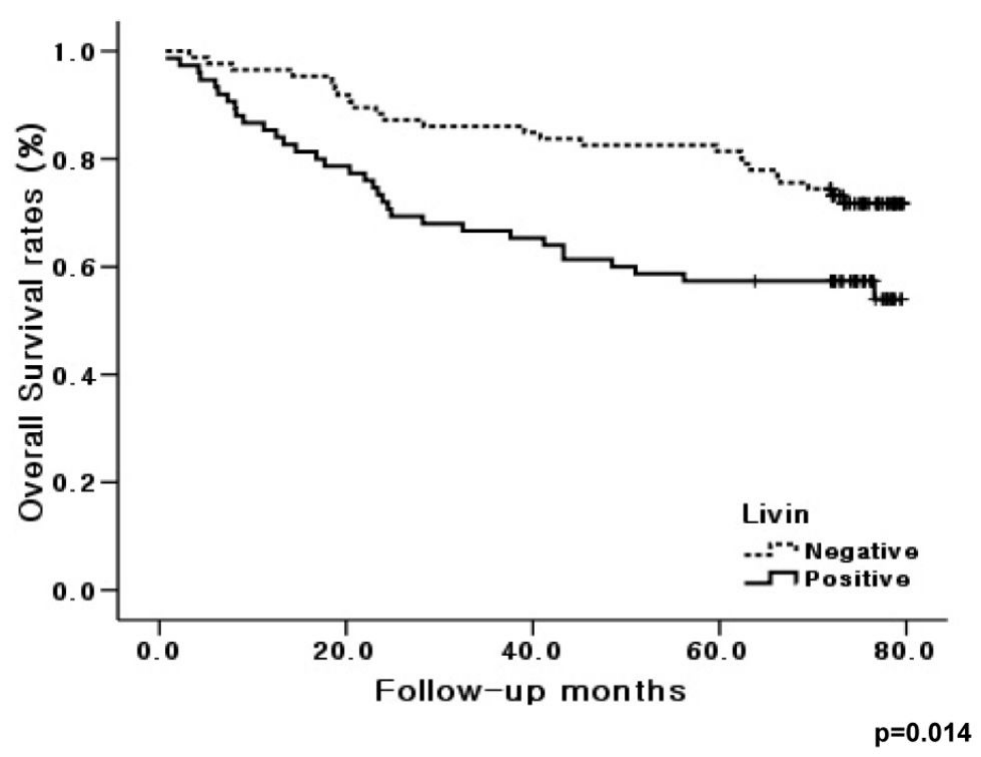
Kaplan-Meier survival curve correlating overall survival with positive expression (solid line) and negative expression (dotted line) of Livin (p=0.014).

To study the prognostic role of Livin in human colorectal cancer progression, we investigated Livin protein expression immunohistochemically in formalin-fixed, paraffin-embedded tissue blocks obtained from 161 patients with colorectal cancer and clinicopathological data, including survival and the correlation between Livin immunostaining and clinicopathological parameters. Livin immunostaining was significantly associated with tumor stage, lymphovascular invasion and lymph node metastasis (p=0.023, p<0.001 and p=0.001, respectively) (Table 2). Moreover, the overall survival of patients with positive Livin immunostaining was significantly lower than that for patients without positive Livin immunostaining (p=0.014) (Figure 8).

**Table 2:** Correlation between Livin expression and the clinicopathological parameters of patients with colorectal cancer.

		Livin expression		
Parameters	Total	Negative	Positive	p-value
	(n=161)	(n=86)	(n=75)	
Age (years)				0.098
<65	71	38	33	
≥65	90	48	42	
Sex				0.381
Male	96	54	42	
Female	65	32	33	
Tumor size (cm)				0.364
< 5	97	49	48	
≥ 5	64	37	27	
Stage				0.023
Ⅰ	20	12	8	
Ⅱ	60	40	20	
Ⅲ	71	31	40	
Ⅳ	10	3	7	
Lymphovascular invasion				<0.001
Negative	103	66	37	
Positive	58	20	38	
Histologic type				0.593
WD	94	50	44	
MD	47	26	21	
PD	7	2	5	
Mucinous	12	7	5	
Signet	1	1	0	
Depth of invasion (T)				0.084
T1	7	1	6	
T2	21	14	7	
T3	125	68	57	
T4	8	3	5	
Lymph node metastasis (N) ( metastasis(N)				0.001
N0	82	54	28	
N1-3	79	32	47	
Distant metastasis (M)				0.125
M0	151	83	68	
M1	10	3	7	

WD, well differentiated; MD, moderately differentiated, PD, poorly differentiated

## Discussion

Apoptosis is a highly regulated process, and various promoters/inhibitors are involved in many steps of this process [9-11]. The IAPs comprise a family of highly conserved cell apoptosis inhibitors with one baculovirus IAP repeat (BIR) domain and a COOH-terminal RING finger domain that suppress apoptosis induced by a variety of stimuli [12]. Livin has been identified as a new member of the IAP family. Livin is overexpressed in tumor tissues and is detected at substantially lower levels or not expressed at all in corresponding normal tissues, suggesting a role for Livin in tumor development and progression [13-16].

The regulation of cell migration, invasion and proliferation is crucial for maintaining cellular homeostasis, and its loss is a principal hallmark of cancer cells. Additionally, cancer cells are typically characterized by increased resistance to apoptosis and cell cycle control [6-8]. Our study showed that knockdown of Livin suppressed tumor cell migration and invasion, and induced apoptosis and cell cycle arrest in human colorectal cancer cells. In contrast, overexpression of Livin using pcDNA3.1 enhanced tumor cell migration and invasion, and inhibited apoptosis and cell cycle arrest. These results suggest that Livin contributes to alter the invasive and oncogenic phenotypes of human colorectal cancer cells.

The anti-apoptotic mechanism of Livin is mediated by direct inhibition of caspases-3, -7, and -9, through its BIR domain and is negatively regulated by the endogenous IAP antagonist SMAC/DIABLO through the COOH-terminal RING finger domain. SMAC/DIABLO binds to the other IAPs including Survivin and XIAP and promote apoptosis [13-16]. In our study, cleaved caspase-3, -7, and PARP expressions were up-regulated in human colorectal cancer cells after Livin knockdown and down-regulated after overexpression of Livin. Survivin protein level decreased, but XIAP and SMAC/DIABLO protein levels were unaltered in response to Livin knockdown. Additionally, Survivin, XIAP and SMAC/DIABLO protein levels were unaltered after overexpression of Livin. Therefore, Livin exerts its anti-apoptotic potential function by directly inhibiting caspase activity in human colorectal cancer cells.

CDKs play a central role in the regulation of cell cycle progression and their activities are regulated by the binding of positive effectors, the cyclins, by negative regulators, the CDKIs, and by phosphorylation and dephosphorylation events [25,26]. The CDKIs are two distinct groups of enzymes. Group 1 is the Cip/Kip family including p21, p27, and p57. Group 2 is the INK family including p15, p16, p18, and p19 [25,26]. Our study showed that knockdown of Livin induced cell cycle arrest by decreasing cyclin and CDKs, and by inducing p27 expression. And overexpression of Livin inhibited cell cycle arrest by increasing cyclin and CDKs, and by decreasing p27 expression. Among the many reported CDKIs, decreased expression of p27 has been described in cancer cells and has also been associated with tumor progression in various human cancers including colorectal cancer [27-29]. Therefore, Livin may contribute to progression of colorectal cancer via cell cycle regulation and p27 blockade.

To explore the potential mechanisms involved in these Livin effects, we studied the effect of Livin on the stimulation of multiple intracellular signaling pathways leading to inhibition of apoptosis and cell cycle progression. The MAPK, phosphatidylinositol-3 kinase (PI3K)/Akt and nuclear factor-kappa B (NF-κB) signaling pathways are involved in many cellular programs such as cell survival and proliferation [30-32]. Livin inhibits apoptosis by regulating JNK1 and PI3K/Akt signaling pathway in lung adenocarcinoma cell line [33,34]. Additionally, Livin regulates prostatic cancer cell invasion through the NF-κB signaling pathway [35]. In our study, phosphorylation of MAPK signaling proteins including ERK1/2, JNK, and p38 was significantly blocked by knockdown of Livin. But, phosphorylation of Akt and NF-κB signaling proteins remained unchanged by knockdown of Livin. Additionally, phosphorylation of MAPK, Akt and NF-κB signaling proteins was not changed by overexpression of Livin. The results suggest that Livin might mostly regulate human colorectal cancer cell behaviors through the MAPK signaling pathway.

Livin is highly expressed in various human cancer types and has also been associated with tumor progression [17-22]. Next, we evaluated Livin expression in human colorectal cancer tissues and paired normal colorectal mucosa of the same patients. We confirmed up-regulation of Livin expression in cancer tissues compared to that in paired normal mucosa at the RNA and protein levels in fresh colonoscopic biopsy specimens. Additionally, Livin expression was increased metastatic lymph node tissues compared to that in non-metastatic lymph node tissues in fresh surgical specimens. These results suggest that Livin has the potential to promote tumor progression.

Cell proliferation and cell death must be properly balanced to maintain tissue homeostasis. However, cancer development and progression result from the imbalance between cell proliferation and cell death, most of which occur through apoptosis [9-11]. Therefore, we evaluated the correlation between Livin expression and tumor cell proliferation or apoptosis in human colorectal cancer tissues. We found no significant difference between Livin expression and tumor cell proliferation. The mean AI value of Livin-positive tumors was significantly lower than the AI of Livin-negative tumors. These *in vivo* results are in accordance with the conclusion that Livin plays a crucial role inhibiting apoptosis in cancer cell lines.

Finally, we documented Livin expression in a well-defined series of human colorectal cancers, including long-term and complete follow-up, with special reference to patient prognosis. Livin expression was significantly associated with tumor stage, lymphovascular invasion, lymph node metastasis, and poor survival. Livin expression is associated with cancer progression and poor prognosis in bladder cancer and neuroblastoma [17,19]. Another report showed that Livin expression is associated with recurrence and presence of lymph node metastasis and overall survival in 61 patients with human colorectal cancer [22]. These results, including ours, suggest that Livin expression may help predict the poor clinical outcome of human colorectal cancer.

Taken together, Livin is associated with tumor progression by increasing tumor cell motility and inhibiting apoptosis in human colorectal cancer and may be useful as a molecular marker for predicting clinical outcomes of patients with colorectal cancer.
